# Xyloglucan Biosynthesis: From Genes to Proteins and Their Functions

**DOI:** 10.3389/fpls.2022.920494

**Published:** 2022-06-02

**Authors:** Jordan D. Julian, Olga A. Zabotina

**Affiliations:** Roy J. Carver Department of Biochemistry, Biophysics and Molecular Biology, Iowa State University, Ames, IA, United States

**Keywords:** polysaccharide biosynthesis, hemicellulose, glycosyltransferase, protein structure, sub-Golgi localization, multiprotein complex

## Abstract

The plant’s recalcitrant cell wall is composed of numerous polysaccharides, including cellulose, hemicellulose, and pectin. The most abundant hemicellulose in dicot cell walls is xyloglucan, which consists of a β-(1- > 4) glucan backbone with α-(1- > 6) xylosylation producing an XXGG or XXXG pattern. Xylose residues of xyloglucan are branched further with different patterns of arabinose, fucose, galactose, and acetylation that varies between species. Although xyloglucan research in other species lag behind *Arabidopsis thaliana*, significant advances have been made into the agriculturally relevant species *Oryza sativa* and *Solanum lycopersicum*, which can be considered model organisms for XXGG type xyloglucan. In this review, we will present what is currently known about xyloglucan biosynthesis in *A. thaliana*, *O. sativa*, and *S. lycopersicum* and discuss the recent advances in the characterization of the glycosyltransferases involved in this complex process and their organization in the Golgi.

## Introduction

Within the plant cell wall, numerous polysaccharides and a group of proteins are involved in one of the strongest natural barriers. These polysaccharides are categorized as either cellulose, hemicellulose, or pectin. The abundant plant hemicellulose xyloglucan (XyG) is the most common hemicellulose in dicots and has been found in numerous monocots (Pauly and Keegstra, 2016). XyG was named for its cellulose-like β-(1- > 4) glucan backbone that is heavily xylosylated. The patterns of XyG xylosylation are found in two forms, XXGG and XXXG, depending on the species. The complexity of XyG varies throughout plants, with various branches composed of xylose (Xyl), galactose (Gal), arabinose (Ara), and fucose (Fuc) monosaccharides, as well as the C6 acetylation of Glc or Gal residues. XyG nomenclature is denoted by single letters, which were introduced by Fry et al. (1993) and have been expanded upon. G represents β-D-Glucose (Glc) with no branching, and X represents a xylosylated Glc, α-D-Xyl-(1- > 6)-β-D-Glc. These Xyl residues can be further branched with Gal, Ara, or another Xyl residue denoted as L, S, and U, respectively. Modifications such as acetylation are denoted with an underlined symbol, such as XXGG, with acetylation of the Glc in the third position. Recent literature has proposed new nomenclature for acetylated XyG branches, but this nomenclature has not been commonly used so far, and as such, this review will utilize the underlined nomenclature (Fry et al., 1993; Tuomivaara et al., 2015). Many additional linkages have been identified, of which most have been compiled by multiple reviews and research articles (Fry et al., 1993; Zabotina, 2012; Schultink et al., 2015; Tuomivaara et al., 2015; Pauly and Keegstra, 2016).

**Figure 1:**
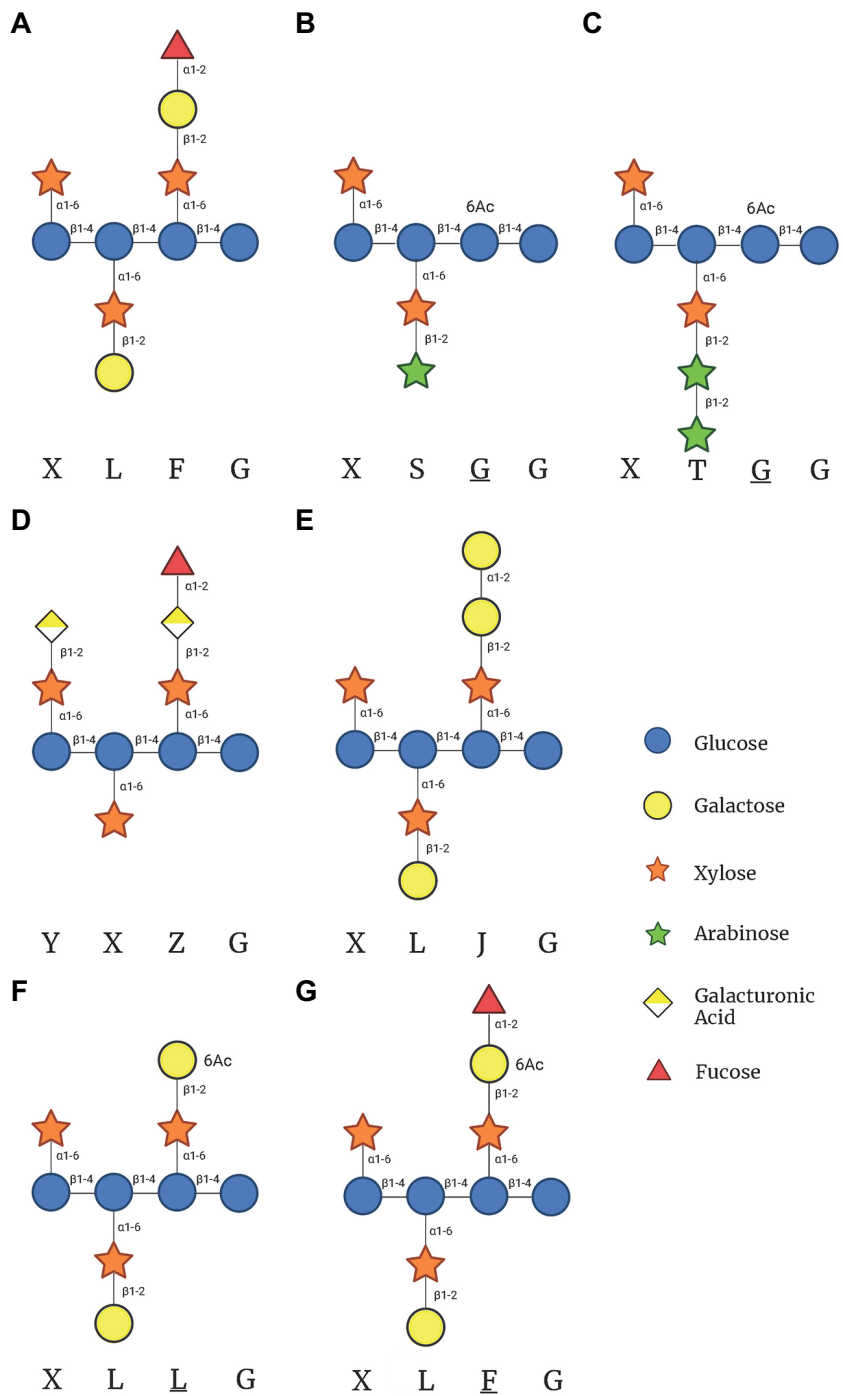
Xyloglucan (XyG) subunits mentioned in this review found within hemicellulose extracts of plant cells walls. **(A)** The common XyG subunit XLFG found in *Arabidopsis* cell walls. **(B)** XyG subunit found in XXGG type XyG from plants such as tomato and rice. **(C)** Diarabinosylated XyG subunit in XXGG type xyloglucan of tomato. **(D)** Fully branched XyG subunit found in *Arabidopsis* root hairs. **(E)** XyG subunit synthesized in the absence of GDP-Fuc in *mur1* mutant plants. **(F)** Gal-6-*O*-acetylated XXXG type XyG after the hypothetical removal of fucose by Axy8. **(G)** Fucosylated XXXG XyG subunit with Gal-6-*O-*acetylation. This figure was created with Biorender.com.

In *Arabidopsis thaliana*, the β-D-Gal-(1- > 2)-α-D-Xyl glycosylation only occurs in the second and third position of the tri-xylosylated pattern resulting in XLXG, XXLG, and XLLG subunits. The third side chain can be extended with an α-L-Fuc *via* an α-(1- > 2) glycosidic linkage [α-L-Fuc-(1- > 2)-β-D-Gal-(1- > 2)-α-D-Xyl] to form XLFG, the most complex naturally occurring XyG subunit within most *Arabidopsis* tissues (Figure 1A; Pauly and Keegstra, 2016). Other species, such as *Oryza sativa*, *Solanum lycopersicum* (rice and tomato), and many types of grasses contain XXGG type XyG in their cell walls. Tomato cell walls lack fucosylated XyG and instead are constituted by mono- (S) and di-arabinosylated (T) XyG side chains with *O*-6-acetylation of the glucan backbone, resulting in XSGG and XTGG (Figures 1B,C; Jia et al., 2005; Schultink et al., 2013; Pauly and Keegstra, 2016). Additionally, a unique subunit of XyG has been found within the roots of *Arabidopsis*, with the first and third branches modified with galacturonic acid (GalA) to form YXYG and YXZG. GalA is the only monosaccharide to be attached to the first Xyl in *Arabidopsis* XXXG type XyG (Figure 1D; Peña et al., 2012).

Synthesis of XyG and most polysaccharides is carried out by glycosyltransferases (GTs). GTs are enzymes that utilize nucleotide-activated sugars such as UDP-Glc, UDP-Xyl, UDP-Gal, GDP-Fuc, UDP-Ara, or UDP-GalA as substrates (donors) and catalyze the transfer of the specific monosaccharides to a specific acceptor. Acceptors have been found to include the four building blocks of life: nucleotides, proteins, lipids, and other glycans, producing glycosylated RNA, glycoproteins, glycolipids, and polysaccharides (Reichmann and Gründling, 2011; Riley et al., 2019; Flynn et al., 2021). GTs have been believed to be very specific in the binding of both donors and acceptors *in vivo*; however, more recent *in vitro* studies have demonstrated that their specificity to be more promiscuous than originally thought (Ohashi et al., 2019; Ruprecht et al., 2020; Ehrlich et al., 2021). GTs have been divided into more than 110 families within the CAZy database,^1^ demonstrating a vast variety between GTs (Drula et al., 2022). Classification of GTs is based on their fold, mechanism, and biochemical pathway. GT-A and GT-B are the most common Rossman folds of GTs, while a third fold has been proposed to have a GT-C fold (Lairson et al., 2008; Chang et al., 2011). Furthermore, GTs can either retain or invert the stereochemistry of the sugar in regard to the donor substrate. For example, most donors that are commonly used exhibit an α-bond. GTs with the retaining mechanism link *via* the same α bond, whereas inverting GTs produce a β-bond attaching the sugar to the acceptor. It is accepted that inverting GTs catalyze reaction using an S_N_2-like mechanism, where an amino acid of the GT acts as the general base to deprotonate the acceptor. The deprotonated acceptor then performs a nucleophilic attack on the donor carbon, displacing the nucleotide pyrophosphate molecule. For retaining GTs, the mechanism is still up for debate. The leading two proposed mechanisms include either a double displacement reaction or a front-sided SN_i_ like displacement mechanism (Gómez et al., 2012; Schuman et al., 2013; Albesa-Jové et al., 2015; Zabotina et al., 2021).

All GTs are membrane proteins classified as either type II membrane proteins or integral membrane proteins, localized to a lipid bilayer within the cell. Type II membrane proteins are single-pass membrane proteins localized to the endomembrane system. Type II GTs have a short N terminal region in the cytoplasm with a single transmembrane domain (TMD) that passes through the Golgi membrane. Inside the Golgi lumen, the GT C-terminal catalytic domain is connected to the TMD through a stem region of variable lengths (Lairson et al., 2008). Integral membrane proteins are multi-pass membrane proteins with 6–8 TMDs embedded into the lipid bilayer. Integral membrane GTs localize to both the Golgi and the plasma membrane (Cocuron et al., 2007; Morgan et al., 2013). XyG biosynthesis is carried out entirely within the Golgi, and once XyG is fully synthesized, it is transported to the cell wall through transport vesicles (Wilkop et al., 2019).

The most recent review on XyG biosynthesis by Pauly and Keegstra (2016) goes into great depth on XyG structural diversity and the known enzymes involved in its biosynthesis. Later that year, FUT1’s structure was reported, the first 3D structure for plant cell wall synthesizing enzyme. Since then, further study has elucidated another structure, XXT1, along with several insights into the other proteins involved in XyG biosynthesis. This review expands the information on XyG biosynthesis to the current standing in the field of XyG biosynthesis and presents the current views and notations about the GTs involved in the process and their mechanisms.

## Proteins Involved in Xyloglucan Biosynthesis

Biosynthesis of XyG is a complex process that involves several GTs that are presumed to be specific towards a particular acceptor structure, e.g., specificity towards the Gal in the third position vs. the second in the XLLG subunit. To date, 13 GTs have been identified which are directly involved in XyG biosynthesis in all *Arabidopsis* tissues, five cellulose synthase-like C proteins (CSLC), five xylosyltransferases (XXTs), two galactosyltransferases (GalT), and one fucosyltransferase (FUT; Faik et al., 2002; Madson et al., 2003; Perrin et al., 2003; Cavalier and Keegstra, 2006; Cocuron et al., 2007; Jensen et al., 2012; Zabotina et al., 2012; Culbertson et al., 2016). All 13 enzymes are localized to the phospholipid bilayer of the Golgi membrane *via* at least one transmembrane domain (TMD). Less studied enzymes, such as galacturonosyltransferases (GalATs), arabinosyltransferases (AraTs), and acetyltransferases (AceTs), have also been demonstrated to be involved in XyG biosynthesis.

### Glucan Backbone Synthesis

CSLC enzymes are members of CAZy GT family 2, a family of inverting integral membrane GTs, and are responsible for the synthesis of the glucan backbone of XyG (Cocuron et al., 2007; Bi et al., 2015). CSLC enzymes are predicted to be integral membrane proteins composed of six TMDs (Davis et al., 2010). The expression of the five CSLC enzymes—CSLC4, 5, 6, 8, and 12—varies throughout different plant tissue (Kim et al., 2020; Tables 1 and 2). CSLC4 and CSLC5 have the highest levels of expression in most vegetive tissues, whereas expression of CSLC6 and CSLC12 is specific to flowers and seeds. Recombinant CSLC4 has been confirmed to synthesize β-glucan in *Pichia pastoris*, while the deletion of all five CSLC GTs in *Arabidopsis* resulted in no detectable XyG in *Arabidopsis* (Cocuron et al., 2007; Kim et al., 2020). The donor substrate for CSLC enzymes, UDP-Glc, is thought to be delivered to the cytoplasmic catalytic domain of the enzymes, which elongates the glucan backbone, moving the polysaccharide through the opening created by the transmembrane helices of the CSLC protein, translocating the glucan chain through the Golgi membrane (Bi et al., 2015). Once the chain enters the lumen of the Golgi, the glucan backbone is then further glycosylated by the other type II membrane proteins described below. It is currently unclear if the CSLC enzymes require an acceptor to begin elongation or if they can synthesize the glucan chain *de novo*.

**Table 1:** Summary of effects of XyG glycosyltransferases (GT) mutants in *Arabidopsis*.

Mutant	Type	Effects on plant	Effects on XyG structure	Citations
*cslc*	Knockout	Small rosettes, shorter inflorescence stems, short root hairs	No detectable XyG	Kim et al., 2020
*xxt1*	Knockout	None	None	Cavalier et al., 2008; Zabotina et al., 2012
*xxt2*	Knockout	None	None	Cavalier et al., 2008; Zabotina et al., 2012
*xxt1 xxt2*	Knockout	Short root hairs, shorter stems, and smaller leaves	No detectable XyG	Cavalier et al., 2008; Zabotina et al., 2012
*xxt5*	Knockout	Shorter root hairs, not as severe as *xxt1 xxt2* double mutant	50% Reduction in IP, higher levels of XXGG subunits	Zabotina et al., 2012
*mur3*	Point mutation	None	Very low levels of Gal and Fuc in third position (XLXG and XXXG only)	Madson et al., 2003; Kong et al., 2015
*mur3*	Knockout	Dwarfed cabage-like growth, short petioles, endomembrane aggregates	No galactosylation of third branch, lacks fucose	Tamura et al., 2005; Kong et al., 2015
*xlt2*	Knockout	None	No galactosylation of second branch	Jensen et al., 2012
*mur3 xlt2*	Knockout	Severely dwarfed plant height	No Gal or Fuc branches	Jensen et al., 2012
*fut1*	Point mutation	None	More than 99% reduction of fucsoylated XyG	Vanzin et al., 2002
*fut1*	Knockout	None	No fucosylation	Perrin et al., 2003
*xut1*	Knockout	Short root hairs	No branches with GalA	Peña et al., 2012
XyBat	Knockout	None	30% Reduction in XyG acetylation	Liu et al., 2016
*axy4/axy4l*	Knockout	None	No Gal *O-*6-acetylation	Gille et al., 2011
*AraTs*	N/A	Not attempted	Not attempted	

**Table 2:** Current understanding of substrate specificity of XyG synthesizing GTs.

GT	Enzyme	Acceptor	Donor	Product	Notes	Citations
CLSC	AtCSLC4/5/6/8/12	G_n_	UDP-Glc	G_n + 1_	Currently unknown if G_1_ can act as an acceptor	Cocuron et al., 2007; Kim et al., 2020
XXT	AtXXT1/2	GGGGGG	UDP-Xyl	XXGGGG		Culbertson et al., 2018
	AtXXT3/4/5	XXGGGG	UDP-Xyl	XXXGGG	Requires XXT1/XXT2 to add first two Xyl	Culbertson et al., 2016
GalT	AtXLT2	XXXG	UDP-Gal	XLXG		Jensen et al., 2012
	AtMUR3	XXXG	UDP-Gal	XXLG		Madson et al., 2003
FucT	AtFUT1	XLLG XXLG	GDP-Fuc	XLFG XXFG		Rocha et al., 2016; Urbanowicz et al., 2017
GalAT	AtXUT1	XXXG	UDP-GalA	YXYG	Only found within the root hairs of *Arabidopsis*	Peña et al., 2012
AraT	SlMUR3	XXXG	UDP-Ara	XXXG	Not observed, only presumed	Schultink et al., 2013
	SlXST1/2	XXXG	UDP-Ara	XXSG	Product found only within *Arabidopsis* mutant cell walls	Schultink et al., 2013
AceT	XyBAT^*^	XXGG XSGG	Acetyl-CoA	XXGG XSGG		Liu et al., 2016; Zhong et al., 2020
	AXY4/AXY4L/XGOATs^**^	XXFG XLFG	Acetyl-CoA	XXFG XLFG		Gille et al., 2011; Zhong et al., 2018

* Includes XyBATs from both *Brachypodium* and *Populus* plants.

** AXY4, AXY4L, and XGOATs are different names proposed for the same AceT homologs. Includes homologs from *Arabidopsis*, rice, and tomato plants.

Besides CSLC proteins, the CAZy GT2 family also includes cellulose synthases (CeS) from plants (CesA) and bacteria (BcsA), the enzymes responsible for the biosynthesis of cellulose, the major polysaccharide within the plant cell wall (Morgan et al., 2013, 2016; Bi et al., 2015; McNamara et al., 2015; Purushotham et al., 2020). Both CesA and BcsA are localized to the plasma membrane and synthesize β-(1- > 4) glucan, a similar product as CSLC enzymes. As mentioned previously, these enzymes have two functions: synthesis of β-(1- > 4) glucan polymers (cellulose) and the translocation of the nascent polymers through the plasma membrane. Synthesis of the glucan polymer is carried out by the cytoplasmic catalytic domain of CeS GTs. The leading hypothesis of catalytic synthesis is that CeS’s catalyze the transfer of Glc from UDP-Glc to the β-glucan polymer through an S_n_2 reaction, inverting the sugar from α to β (Morgan et al., 2013). The elongated polymer is then shifted to empty the catalytic site, translocating through the plasma membrane (Bi et al., 2015; Morgan et al., 2016). Translocation of the acceptor is carried out by three conserved features: a QxxRW motif, a finger helix with a TED motif, and a gating loop. The conserved QxxRW sequence is located near the channel’s entrance and is responsible for the stabilization of the acceptor through π and hydrogen bonds (Morgan et al., 2016; Zimmer, 2019). Additionally, the finger helix’s TED motif interacts with the acceptor polysaccharide by forming hydrogen bonds with three hydroxyls of terminal Glc. After the transfer of Glc (N) to the acceptor, the finger motif shifts from the previous terminal Glc (N + 1) back into the catalytic domain to interact with the newly added Glc (N). An incoming UDP-Glc along with the gating loop then pushes the finger motif up into the transmembrane domain, shifting the glucan polysaccharide by one sugar residue. The rest of the Glc chain is stabilized by numerous π interactions within the channel, with only strong hydrogen bonds interacting with the terminal Glc.

This same translocation may be shared by CSLC enzymes as well, but within the Golgi membrane instead of the plasma membrane. Utilizing the new revolutionary software AlphaFold 2.0 to predict CSLCs’ structure, we observed that CSLCs are highly homologous to the known BcsA structure (PDBID 4P00; Jumper et al., 2021). The predicted structures mentioned in this review (besides AraTs) are publicly available on websites such as UniProt^2^ for download, and alignment can be carried out by PyMol modeling software (Schrödinger and DeLano, 2020). AlphaFold is also publicly available for free, with recent updates including a multimer prediction function, although the software can be demanding and require sophisticated hardware. In the highly homologous structures of CSLCs and CeS, CSLCs share the QxxRW motif with CeS enzymes but lack the TED motif, instead encoding a VED motif. Structural prediction of CSLCs aligns the VED motif of the CSLC GTs with the TED motif from BcsA. A T341V mutation of BcsA or CeSA has never been attempted, so it is challenging to predict how the hydrogen bonding would change the function. However, mutation of both Glu and Asp residues within the TED motif have resulted in reduced cellulose content (Kumar et al., 2018). Homology and computational modeling provide insightful hypotheses for the CSLC mechanisms and structure, while the experiments that have been carried out with CeS GTs provide an excellent model for future studies on CSLCs.

*Arabidopsis* lines lacking a single CSLC protein did not display any noticeable phenotypes. Combinations of *cslc* knockouts resulted in various phenotypes, including smaller rosettes, shorter inflorescence stems and short root hairs (Kim et al., 2020; Table 1). Digestion of the mutant *cslc45812* cell wall with driselase, a cocktail of glycosyl hydrolases lacking α-(1- > 6) xylosidase, resulted in no detectable isoprimeverose [IP; Xyl α-(1- > 6) Glc], resembling that of the *xxt1 xxt2* double mutant described below.

### Xylosylation of the Glucan Backbone

XXTs have been more extensively studied compared to other XyG synthesizing GTs aside from FUT1. In *Arabidopsis*, five XXTs from the retaining CAZy GT family 34 have been identified. XXTs appear to be biochemically redundant, catalyzing the xylosylation of the first three positions in the XXXG subunit. Reverse-genetic studies have demonstrated that the *Arabidopsis* double knockout mutant *xxt1 xxt2* did not produce any detectable XyG, which was confirmed by the lack of IP released by driselase (Cavalier et al., 2008; Zabotina et al., 2012). Knockout of *xxt5* alone significantly reduced levels of detectable IP by 50%, while the altered XyG patterns displayed higher levels of XXGG subunits and lower levels of the typical XXXG subunits (Zabotina et al., 2008, 2012). Similar results were observed when the *xxt5* mutation was paired with either *xxt1* or *xxt2*, suggesting that XXT5 was also required for normal XyG biosynthesis alongside XXT1 and XXT2 *in vivo* (Zabotina et al., 2012). Furthermore, the *xxt1 xxt2* double mutant plants had initial growth defects resulting in shorter stems and smaller leaves (Table 1), although this phenotype disappeared as the plant matured, resembling wild-type *Arabidopsis* (Cavalier et al., 2008; Zabotina et al., 2012). These double mutants also have root hairs that are much shorter, with a rounded structure, as compared to the long, thin wild-type *Arabidopsis* root hairs. The *xxt5* single mutant also had the same root hair phenotype although not as pronounced.

XXT1 is the only XXT structurally characterized to date, which was the second structure of cell wall synthesizing GTs to be described, following the characterization of FUT1. XXT1 was crystallized as a homodimer (PDBIDs: 6BSU, 6BSV 6BSW) which has a GT-A fold (Culbertson et al., 2018). For structural characterization, truncated XXT1 was expressed in HEK293F cells with amino acids 45–460, removing its TMD. Each dimer subunit was structurally oriented in opposite directions, meaning that the dimer likely acts on two separate glucan chains rather than both interacting with the same chain. Mechanistically, XXT1 binds to the donor UDP-Xyl and a β-(1–4) glucan chain acceptor molecule of at least 3 Glc in length, which is the proposed minimum required to be active (Culbertson et al., 2018; Zhong et al., 2021). Like most GT-A folded GTs, XXTs require the metal cofactor Mn^2+^ to be catalytically active, which is coordinated by the DXD motif in XXT1’s binding site (Culbertson et al., 2018). Cocrystalization of UDP-Xyl with XXT1 failed to reveal any bound UDP-Xyl. Instead, UDP was bound in its place, requiring computer modeling to simulate Xyl interactions. This modeling of UDP-Xyl suggested that XXT1’s dominant interactions with the donor substrate occur *via* the sugar molecule rather than the high-energy UDP. These models also suggest that Phe 203 interacts with C5 of the Xyl sugar, which would presumably impair binding to hexose sugars, increasing affinity for UDP-Xyl. The acceptor molecule, cellohexaose, is bound by XXT1 through a mix of hydrogen bonding of the inner for Glc molecules, with weaker water and hydrophobic interactions stabilizing the end Glc sugars (Glc 1 and Glc 6). These interactions orient the C6-hydroxyl of the fourth Glc into the active site of XXT1. As stated previously in this review, the mechanism for retaining GTs is still debated. Of the two prominent theories, double displacement and SN_i_-like displacement, evidence suggests that XXT1 favors an SN_i_-like mechanism. Likely candidate residues for double displacement included two Asp residues that were positioned a large distance (>5 Å) from the anomeric carbon of UDP-Xyl. Mutations of these two Asp to Asn impaired XXT1’s activity, but did not prevent the xylosylation of cellohexaose. G319, the likely residue involved in the SN_i_-like displacement mechanism, was also positioned a significant distance (4.7 Å) from the anomeric carbon. However, a G319A mutation resulted in significantly less activity, ~40% less activity than the double Asp mutant normalization to wild-type XXT1 activity (Culbertson et al., 2018).

Structural characterization of XXTs allowed Culbertson et al. (2018) to propose the N + 2 rule for glucan backbone xylosylation in *Arabidopsis*. The N + 2 rule states that XXTs that fall into this category—XXT1 and XXT2—have steric hindrance preventing the xylosylation of the N + 2 position on the glucan backbone after the N position has already been xylosylated. This steric hindrance is hypothesized to be caused by I391 in both XXT1 and XXT2. Both sequence alignment and structural prediction software such as AlphaFold predict that XXT3, XXT4, and XXT5 encode a G392 in that space instead, providing adequate capacity for the Xyl residue at the N position to fit within the binding cavity of these XXTs (Culbertson et al., 2018).

*In vitro* biochemical assays have confirmed the activity of XXT1, XXT2, XXT4, and XXT5, with varying rates of catalysis and different patterns of xylosylation of cellohexaose, the acceptor molecule used in most XXT *in vitro* assays (Culbertson et al., 2016, 2018; Zhong et al., 2021). XXT1 and XXT2 are kinetically similar and have been shown to synthesize XGGGGG, XXGGGG, XXXGGG, and even XXXXGG patterns in biochemical assays (Culbertson et al., 2016, 2018; Zhong et al., 2021; Table 2). XXT4 and XXT5 have been shown to be significantly slower, requiring a higher enzyme concentration and appear to only synthesize the XGGGGG and XXGGGG pattern in *in vitro* studies (Culbertson et al., 2016; Zhong et al., 2021). Zhong et al. (2021) showed that XXT1/XXT2 are able to add up to four Xyl residues to cellohexaose in *in vitro* assays and proposed that XXT1 and XXT2 are solely responsible for *in vivo* xylosylation of XyG.

**Figure 2:**
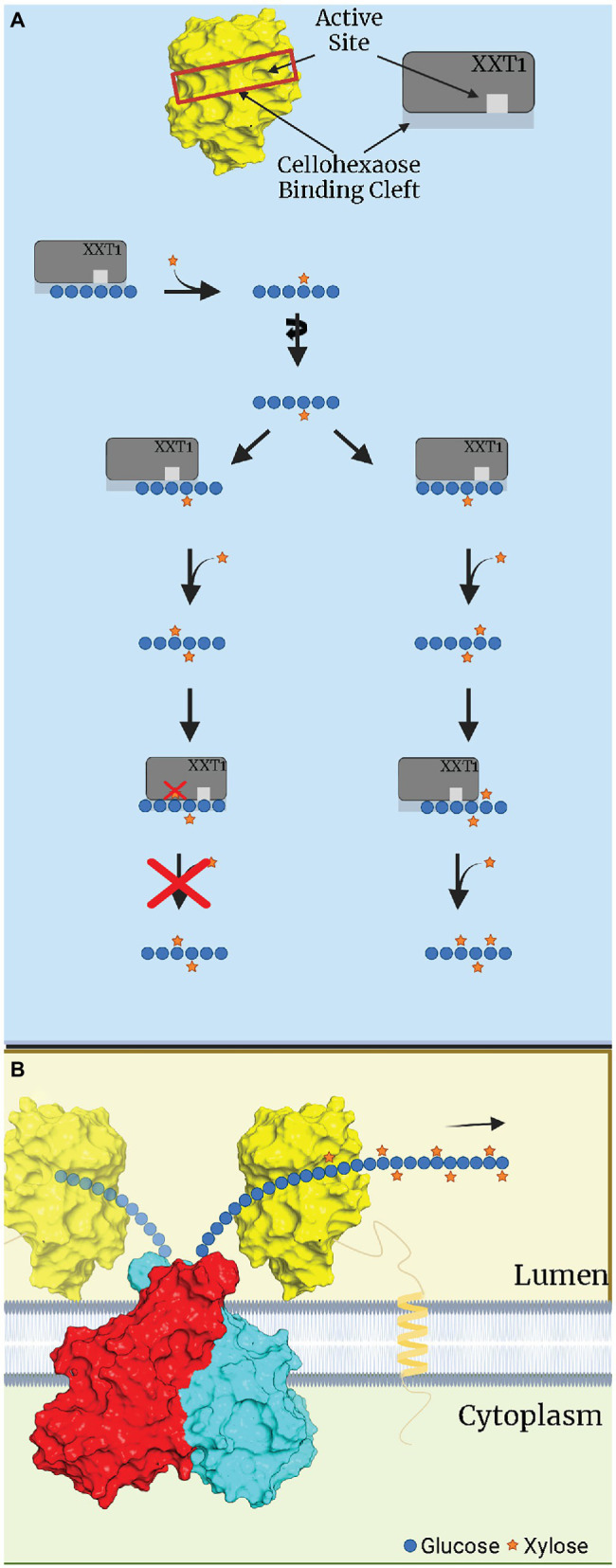
Hypothetical depiction of xylosylation of a glucan substrate by XXT1. **(A)** *In vitro* xylosylation of cellohexaose by XXT1. XXT1 mono- and **FIGURE 2 | **dixylosylates cellohexose without significant steric restriction, resulting in XXGGGG. Once cellohexaose is dixylosylated, XXT1 can accommodate the XXGGGG acceptor by binding it in the proper position lacking steric hinderance, e.g., all Xyl attached earlier are localized outside the binding cleft of XXT1. XXT1 can trixylosylate and tetraxylosylate the cellohexaose substrate due to motional freedom of both XXT1 and the glucan acceptor in solution. **(B)** *In vivo* xylosylation by XXT1 within the Golgi lumen. The glucan backbone is constantly elongated by a CSLC dimer (red, cyan), which is then xylosylated by XXT1 (yellow) attached to membrane *via* transmembrane domain (TMD). The TMD/stem region limits mobility of XXT1, while the glucan backbone is elongated and moves only in one direction, depicted by an arrow. The glucan backbone’s rotational movement is also limited by being bound to CSLC while elongation occurs. CSLC structure was predicted by AlphaFold, while for XXT1 the crystal structure was used (PDBID:6BSW). This figure was created with Biorender.com.

Culbertson et al. (2018) proposed an alternative hypothesis, suggesting that the addition of three consecutive Xyl residues is likely due to the ability of truncated XXT1 and XXT2 to differently bind the glucan oligosaccharide in solution, shifting and rotating the acceptor glucan chain to prevent the previously attached Xyl from being placed in restrictive orientations (Figure 2A; Culbertson et al., 2018). On the contrary, full-length XXTs are attached to the plant Golgi membrane *via* their TMD and likely do not have such motional freedom as truncated XXTs in solution. Within the Golgi, XXTs must continuously xylosylate a constantly elongating glucan chain synthesized by CSLCs. This imposes limitations on both the rotational movement and direction the glucan acceptor moves in the binding site of XXTs, limiting the XXTs’ ability to bind in different positions along the glucan chain (Figure 2B). Such limitations in both the glucan chain interactions with XXTs and the XXTs’ own restricted motions would prevent XXT1 and XXT2 from accommodating the glucan chain with XXGG pattern of substitution, as the first position (N+2) Xyl residue would be blocked by the side chain of the I391 (Culbertson et al., 2018). Thus, the results obtained from structural and reverse-genetic studies suggest a different hypothesis about the mechanism of xylosylation of the XyG backbone by XXTs within the Golgi lumen. It is likely that XXT1 and XXT2 are responsible for adding Xyl to the first two consecutive Glcs, synthesizing XXGG patterns. Whereas XXT5 and its homologs—XXT3 and XXT4—finish xylosylation by adding the third Xyl, completing the XXXG pattern in *Arabidopsis* XyG (Table 2; Culbertson et al., 2018; Zabotina et al., 2021).

Another study also using *in vitro* assays demonstrated that XXT1 is capable of using multiple activated sugars as donor substrates: UDP-Glc, UDP-Gal, and UDP-Ara in addition to UDP-Xyl (Ehrlich et al., 2021). It is worth to note that the rates of XXT1 catalysis using other nucleotide sugars were significantly lower in comparison to UDP-Xyl. Although *in vitro* enzymatic assays demonstrate the tetraxylosylation of sequential glucan residues and the ability to utilize different donor substrates (Zhong et al., 2021), there is no experimental evidence of this occurring *in vivo*. This suggests that there is control of XyG patterns synthesized *in vivo*, which can either be through enzyme substrate specificity or donor substrate availability in the Golgi lumen.

### Galactosylation of Xylose in XyG

To date, only two galactosyltransferases (GalT) have been shown to be involved in *Arabidopsis* XyG biosynthesis, MUR3 and XLT2. XyG galactosylation occurs to the second and third positions of the XyG subunits. Both are members of the inverting CAZy GT family 47. These two GalTs have high sequence homology and have highly homologous structures predicted by AlphaFold. Both GalTs seem to be highly selective towards a specific Xyl residue in the XXXG subunit of *Arabidopsis* XyG. The mutation of *mur3* leads to a lack of both galactosylation and fucosylation of the third Xyl residue, resulting in XLXG (Madson et al., 2003). Recombinant expression in *Pichia pastoris* showed activity of MUR3 after incubation with XXXG/XLXG XyG as an acceptor and radioactive UDP-Gal as the donor (Madson et al., 2003). XLT2 is believed to galactosylate the second Xyl residue, forming XLXG. Analysis of digested XyG from *xlt2* knockout *Arabidopsis* plants displayed a lack of detectable XLXG or XLFG subunits (Jensen et al., 2012). This leads to the conclusion that galactosylation of both Xyl residues of *Arabidopsis* XyG is carried out by separate GalTs that are highly specific to the position of the Xyl residues in the acceptor molecules (Table 2). Both GalTs have been expressed in HEK293S cells, while MUR3 has been expressed in *Pichia*, but as of this review have yet to have activity and specificity reported from *in vitro* assays (Prabhakar et al., 2020).

Aside from changes in XyG, mutant *mur3* lines with a point mutation lacked any noticeable phenotype, while still having low levels of galactosylation and fucosylation of XyG. Mutants with complete deletion of *mur3* resulted in a dwarf cabbage-like phenotype, short petioles and endomembrane aggregates, while some lines were shown to be more salt sensitive (Table 1; Madson et al., 2003; Tamura et al., 2005; Li et al., 2013; Kong et al., 2015). Interestingly, overexpression of *xlt2* resulting in higher levels of XLXG subunits suppressed these phenotypes (Kong et al., 2015). Additionally, double mutants *xxt2 mur3* and *xxt5 mur3* also recovered from these phenotypes, resembling wild-type plants. Cell wall analysis of these plants detected higher levels of XLXG subunits than that of the single *mur3* mutant indicating that these phenotypes are a result of shorter XyG branches.

### Fucosylation of Galactose

Only one XyG synthesizing fucosyltransferase, FUT1, is encoded by *Arabidopsis* and is a member of the inverting GT37 family (Cicéron et al., 2016; Rocha et al., 2016). FUT1 catalyzes the fucosylation of Gal in the third branch of the XLLG and XXLG subunits using GDP-fucose as a donor substrate. Mutations in the FUT1 protein (*mur2*) in *Arabidopsis* led to a reduction in fucosylation of the cell wall by 50%, while only 2% of XyG was fucosylated (Table 1; Vanzin et al., 2002). The mutation in *mur2 Arabidopsis* plants was identified to be D550N mutation, replacing a carbocyclic group with a carboxamide that can potentially be N-glycosylated. From the crystal structure, FUT1 amino acid 550 is found located in the extra C-terminal region, which is involved in the binding of the XyG acceptor substrate (Rocha et al., 2016). FUT1 was the first plant cell wall synthesizing GT to be structurally characterized. For structural analysis, two independent groups expressed FUT1 in two different eukaryotic cells, insect and HEK293S, which were truncated at the 68^th^ and 80^th^ amino acids, respectively, to remove its TMD (Cicéron et al., 2016; Rocha et al., 2016; Urbanowicz et al., 2017). Each truncation of FUT1 formed a homodimer in solution and crystalized as a homotetramer (Rocha et al., 2016; Urbanowicz et al., 2017). Similarly, full-length FUT1 has been reported to also form a homodimer *in vivo* (Chou et al., 2015). Although crystal structures were unable to capture FUT1 bound to GDP-Fuc, GDP was successfully bound (Rocha et al., 2016, Urbanowicz et al., 2017). Rocha et al. (2016) proposed that D300 of FUT1 acts as a catalytic base for an S_N_2 reaction, confirmed by the reported mutation of D300A, which resulted in a lack of activity. Alternatively, Urbanowicz et al. (2017) concluded that D300 is not actually a catalytic base; rather water is necessary for a two-step S_n_2 reaction (Urbanowicz et al., 2017). This was concluded because 1- a lack of residue conservation by other presumed homologs; 2- a large distance of 5 Å between D300 and the XXLG’s Gal-O acceptor; 3- a repetitive presence of a water molecule within the active site in all reported crystal structures, including those from Rocha et al. (2016); Urbanowicz et al. (2017). Mutation of D300A in the later study led to reduced kinetics but failed to completely inactivate FUT1 activity, contradicting the earlier results reported by Rocha et al. (2016). It should be noted that both studies differed in their activity assays, with Rocha et al. (2016) utilizing a radioactive C_14_ labeled assay and Tamarind XyG as an acceptor. Urbanowicz et al. (2017) utilized the commercially available GDP-Glo kit and used highly purified XXLG fragments as the acceptor substrate. Urbanowicz et al. (2017) also suggested that the previous study of Rocha et al. (2016) may not have used sufficient concentration of FUT1 enzyme, which resulted in a lack of activity seen in D300 and other impaired mutants. Although both studies present plausible mechanisms, it remains unclear which mechanism FUT1 follows. Further comprehensive studies with improved dynamic analysis such as Cryo-EM is likely needed to further clarify the mechanism of fucosylation catalyzed by FUT1.

Recent biochemical studies suggest that FUT1 may be less specific than initially suggested. While FUT1 has been observed to only glycosylate the third branch in the XXXG subunit, its substrate recognition has been shown to be versatile. Besides fucosylation, FUT1 also may act as a GalT in the absence of GDP-Fuc. *In vitro* assays demonstrated that FUT1 transfers Gal to an XLLG or XXLG substrate at the third position, resulting in an XLJG or XXJG product, respectively (Figure 1E; Ohashi et al., 2019). While capable, the activity was 1/3 that of a GDP-Fuc substrate, indicating that FUT1 has a higher affinity for GDP-Fuc rather than for GDP-Gal. The above XLJG product is observed in a mutant strain of *Arabidopsis*, *mur1*, which also produced an XLJG product (Zablackis et al., 1996). The *mur1* mutation has been linked to an epimerase (GDP-D-Mannose-4,6-Dehydratase 2) responsible for the *de novo* synthesis of GTP-Fuc, leading to defects in all cell wall polysaccharides that contain Fuc (Bonin et al., 1997). Additionally, when the *mur1* mutant was crossed with *mur2* mutant, a lack of both galactosylation and fucosylation of XyG was observed, providing further evidence that the galactosylation was carried out by FUT1 due to a lack of GDP-Fuc availability (Vanzin et al., 2002). Acceptor recognition may also be flexible as FUT1 recognizes GalA in place of Gal. XyG in root hairs with GalA residues [β-d-Gal*p*A-(1 → 2)-α-d-Xyl*p*-(1 → 6)-β-d-Glc*p*; denoted Y; XXYG] are still fucosylated, resulting in an XXZG subunit (Figure 1C) [α-l-Fuc*p*-(1 → 2)-β-d-Gal*p*A-(1 → 2)-α-d-Xyl*p*-(1 → 6)-β-d-Glc*p*; denoted J] (Peña et al., 2012).

### Arabinosylation of XXGG Type Xyloglucan

**Figure 3:**
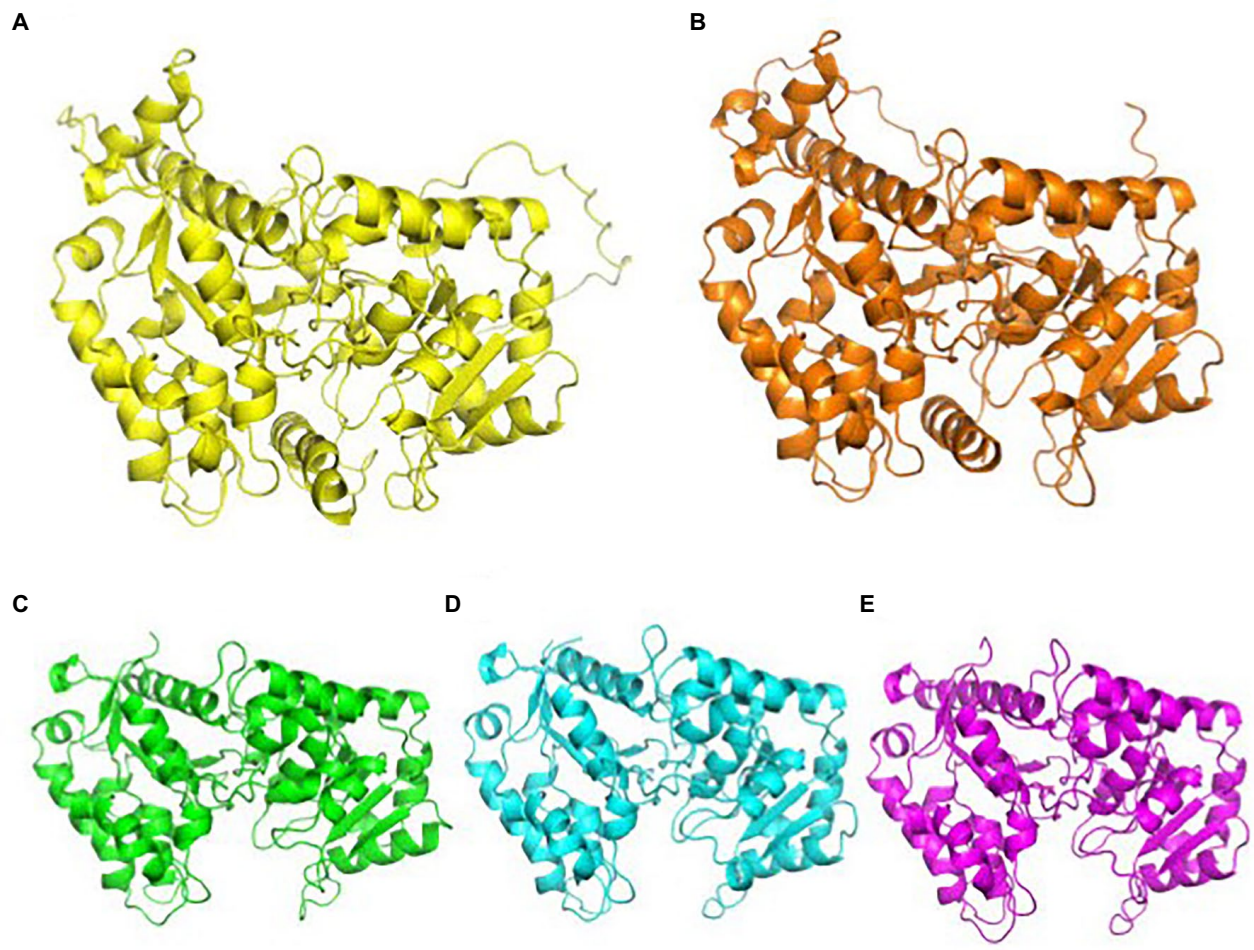
AlphaFold predictions of CAZy family 47 GalTs and AraTs. GalTs are readily available at uniport.org, while AraTs were generated by AlphaFold for this review. **(A)** Structural prediction of AtMUR3, color: yellow. **(B)** Structural prediction of SlMUR3, color: orange. **(C)** Structural prediction of AtXLT2, color: green. **(D)** Structural prediction of SlXST1, color: cyan. **(E)** Structural prediction of SlXST2, color, magenta. The TMD and stem regions were removed for clarity. AlphaFold’s prediction of the TMD and stem regions of these proteins are highly inaccurate, as computation software still struggles with transmembrane proteins. Images were aligned and generated using PyMOL.

XyG arabinosylation is not well characterized, as it does not naturally occur in *Arabidopsis*. XyG arabinosylation occurs in the second position in XXGG type XyG, although the third position is arabinosylated in mutant plants that have XXXG type XyG. Arabinosylated XyG is prominently found in many Solanaceous plants, such as tomatoes and potatoes, which synthesize the XXGG pattern of XyG. In these species, the dominant subunits of XyG are found as XXGG, XSGG, and LSGG with acetylation of the third Glc in the glucan chain (Figure 1B; Jia et al., 2003). Similar to MUR3 and XLT2, arabinosyltransferases (AraT) are members of the GT47 family and are sequentially and functionally homologous to GalTs. Predictions from AlphaFold indicate that GalTs and AraTs have high structural homology. These structures are not available on uniprot.com and have been provided in this review (Figure 3). Instead of the galactosylation of the second and third XyG side chains, homologs of MUR3 and XLT2 within Solanaceous plants are suspected to transfer L-arabinofuranose rather than L-galactopyranose (Madson et al., 2003). Three putative AraT homologs in tomatoes were identified and transfected into *mur3 xlt2 Arabidopsis* mutants (Schultink et al., 2013). Independent expression of SlMUR3 (homolog to MUR3), SlXST1, and SlXST2 (both homologous to XLT2) in *mur3 xlt2 Arabidopsis* mutants resulted in XyG with XXSG structure. XST1 mutants produced higher levels of XXSG XyG than other transfected AraT homologs, while the SlMUR3 expressing plants did not produce any XXSG (Table 2; Schultink et al., 2013).

The effect of AraTs on tomato cell walls and growth is currently unknown. It is suspected that they arabinosylate the second position in the XXGG subunit to form XSGG, but it is unknown why tomato plants encode three separate AraTs for this task. Expression of AraTs in dwarfed *mur3 xlt2* mutant *Arabidopsis* plants resulted in comparable plant height to wild-type *Arabidopsis* plants. Due to predicted high structural homology between GalTs and AraTs, most likely, the former can easily occupy the same position in the XyG synthesizing complex in *Arabidopsis* Golgi to be discussed below, as they likely share similar recognition mechanisms. This will be interesting to investigate in the future, as the activity of these AraT have yet to be demonstrated.

### Galacturonic Acid Substitution of Galactose

Xyloglucan-specific Galacturonosyltransferase1 (XUT1) is another predicted homolog to GalTs and is also a member of the CAZy family 47. XUT1 has only been found within the root hairs of *Arabidopsis* (Jensen et al., 2012; Peña et al., 2012). Previously reported as GT16 through transcriptome analysis, the activity of XUT1 has not been reported (Jensen et al., 2012). The current evidence suggests it transfers GalA to the first and third Xyl residues in the XyG subunit, resulting in the acidic YXXG, XXYG, and YXYG XyG subunits found only within the roots of *Arabidopsis* plants (Figure 1D, Table 2). Once XUT1 was deleted, XyG within the roots resembled that of XyG from vegetative tissue (XLFG), lacking acidic subunits. Plants with the *xut1* mutation also had significantly shorter root hairs than wild type *Arabidopsis* plants when grown on nutrient media (Table 1). *In vitro* activity of XUT1 has yet to be reported, although recombinant expression has been successful (Prabhakar et al., 2020). Interestingly, the GalA residue in the third chain can still be fucosylated in place of the typical Gal, resulting in the XXZG and YXZG XyG subunits (Figure 1D; Peña et al., 2012; Urbanowicz et al., 2017).

### *O*-Acetylation of XyG

Currently, there are two distinct forms of *O*-acetylation in XyG molecules: acetylation of Gal residues and 6-*O*-acetylation of the glucan-backbone. *O*-Acetylating enzymes are classified into three families: Trichome-Birefringence-Like (TBL), Reduced-Wall-*O*-Acetylation (RWA), and a family represented by the enzyme Altered Xyloglucan 9 (AXY9; Schultink et al., 2015; Pauly and Ramírez, 2018). Members of each family are likely involved in the *O*-acetylation of several polysaccharides within the cell wall, including mannans, xylan, and XyG. Members of both TBL and AXY9 families are predicted to be type-2 membrane proteins, with their C-terminal catalytic domains within the Golgi lumen, similar to the GTs described above. Alternatively, RWA proteins are predicted to be integral membrane proteins, with up to 10 predicted transmembrane domains. These RWA proteins may act as transporters for unknown donors of acetyl groups rather than polysaccharide-specific acetyltransferases, although it is not confirmed (Pauly and Ramírez, 2018).

*O*-acetylation of Gal residues in the XyG side chain has been found in several organisms with XXXG type of XyG. XyG from sycamore and *Arabidopsis* cell walls were found to exhibit predominantly 6-*O-*monoacetylation of Gal residues, but 3-*O* and 4-*O-*monoacetylation and 3,4- and 4-6-di-*O*-acetylation has been observed (York et al., 1988; Zhong et al., 2018). Two proteins have been linked to the *O-*acetylation of Gal in *Arabidopsis* XyG: AXY4 and AXY4L. Single mutants lacking each gene indicated that AXY4 is responsible for *O*-acetylation in sprouting plants while AXY4L is active in seeds (Table 2; Gille et al., 2011). *O-*acetylation only occurs on the third Gal in the XXLG subunit, resulting in XXLG-like subunits (Figure 1F). XyG extracted from *Arabidopsis* cell walls exhibit acetylation of Gal residues in XLXG/XXLG, XLLG, XXFG and XLFG subunits. *In vitro* activity assays of AtAXY4, AtAXY4L, and homologs from *Populus trichocarpa* referred to as XGOATs, demonstrated that *O-*acetylation of Gal only occurred in subunits that had been pre-fucosylated, i.e., XXFG and XLFG. Gal residues in subunits lacking fucosylation, XLXG, XXLG, and XLLG, were not *O*-acetylated. This led Zhong et al. (2018) to propose that AXY4 and AXY4L are both specific towards Gal residues that have been fucosylated. They suggest that after the XyG is transported to the cell wall, AXY8, a fucosidase localized to the apoplast, defucosylates both acetylated and non-acetylated XyG, resulting in the reported XLLG and XXLG subunits within the cell walls (Figures 1F,G; Table 2; Zhong et al., 2018). Other putative acetyltransferases, such as AXY9, have been shown to be involved in the *O-*acetylation of multiple hemicelluloses, as knockout resulted in an overall reduction in cell wall acetylation. So AXY9 has been proposed to have an independent mode of acetylation from that of AXY4 and AXY4L and may function upstream of the two enzymes (Schultink et al., 2015). *In vitro*, AXY9 displayed minimal activity when incubated with mixed XyG acceptors, similar to the activity of AXY4 and AXY4L incubated with non-fucosylated XyG acceptors. This suggested that AXY9 is not specific for XyG acetylation, but still acts as an acetylesterase (Pauly and Ramírez, 2018; Zhong et al., 2018).

The glucan backbone is also directly *O-*acetylated at the C6 carbon of the third Glc in XXGG type XyG, resulting in XXGG (Jia et al., 2005). *O-*acetylation of the glucan chain has only been observed in species that produce the XXGG pattern of XyG, such as tomatoes. Glucan backbone acetylation has been linked to the protein XyBAT1, first discovered in *Brachypodium distachyon* (Liu et al., 2016). Upon the knockout of XyBAT1 in *Brachypodium*, there was a mass shift in the XyG profile, resulting in a new pattern of xylosylation resembling that of XXXG type XyG. Additionally, *xybat1* mutant plants displayed a 30% reduction in XyG acetylation (Table 1), while a reduction in other hemicellulose acetylation was not detected, indicating XyBat1 is specific for XyG acetylation. Using enzymatic assays, several homologs of XyBAT1 from rice and tomato have also been confirmed as acetyltransferases that are specific for the β-glucan backbone of XyG (Zhong et al., 2020). Upon expression of BdXyBAT1 in *Arabidopsis mur3 xlt2* mutant plants, the growth defect was rescued while the XyG mass-profile changed again with a low level of XyG resembling that of the *Brachyprodium* XXGG type (Liu et al., 2016). Furthermore, the dwarfism phenotype caused by the deletion of XLT2 and MUR3 was rescued by the expression of BdXyBAT1, resulting in the formation of acetylated XyG (Liu et al., 2016). On the other hand, rice XyBAT (OsXyBAT6) was expressed in wild-type *Arabidopsis*, which led to a severe growth defect phenotype. Cell wall analysis revealed that XyG from OsXyBAT1 transfected plants had subunits that were vastly different, with rarely detected patterns such as XG, FG, XFG, and more (Zhong et al., 2020). It appears that acetylation of the glucan backbone may compete with xylosylation and potentially be responsible for the lack of xylosylation on the third Glc in XXGG subunits in grass and tomato XyG. Further study is necessary to understand the specificity of each acetyltransferase and the XyG biosynthesis process in regard to acetylation.

## Complex Formation and Localization of XyG Synthesizing Enzymes Within the Golgi

Like most polysaccharide synthesizing GTs, GTs involved in XyG synthesis are localized to the Golgi. The specific localization of GTs within the Golgi can promote the formation of multiprotein complexes differentially localized in the Golgi cisternae. Separation of Golgi stacks (*cis*, *medial*, and *trans*) using free-flow electrophoresis (FFE) paired with immunodetection of XyG using carbohydrate specific antibodies and immunogold labeling depicted the localization of XyG molecules throughout the Golgi stacks of *Arabidopsis* have provided insight into sub-Golgi localization (Parsons et al., 2019). High levels of only xylosylated XyG (XXXG subunit) were detected within the *cis*-cisternae with limited detection of low levels of galactosylated and fucosylated XyG. Xylosylated XyG was found to be similarly distributed throughout the *medial*-Golgi, with a moderate increase in galactosylated and fucosylated forms of XyG substitution. Finally, within the *trans*-Golgi, xylosylated XyG levels decreased slightly, while high levels of XLFG and XLLG were detected (Parsons et al., 2019). Independently, heterologous expression of GFP-tagged *Arabidopsis* GTs XXT1, MUR3, and FUT1 using tobacco BY-2 suspension-cultured cells demonstrated somewhat similar results of localization to that of the XyG polysaccharides (Chevalier et al., 2010). In the latter study, immunogold electroscopic microscopy showed XXT1 was localized to both *cis*- and *medial*-cisternae, where xylosylated XyG was most abundant. MUR3 and FUT1 localized to the *medial*- and *trans*-Golgi, where higher levels of completely branched XyG was localized. It is currently unknown what controls the localization of GTs in the Golgi, but it has been proposed that the GTs form a dynamic multicomplex in which proteins interact and separate in different cisternae (Chevalier et al., 2010; Zabotina et al., 2021). As to localization control, small peptide motifs within the N-terminals of GTs may be responsible for transportation and localization throughout the Golgi, localizing each to the correct cisternae. While a signaling peptide has yet to be confirmed for GTs and many plant localized Golgi proteins, yeast and mammalian cells have been thoroughly investigated and suggest plants may share the same machinery (Zhang and Zabotina, 2022).

Larger order complexes are quite common in many biosynthetic pathways, and some earlier evidence suggests XyG biosynthesis is not an exception. Several examples of the formation of complexes among GTs involved in the synthesis of different polysaccharides have been reported (Zabotina et al., 2021 and citations within). Some studies have given compelling evidence that some of these protein complexes may even be required for efficient biosynthesis (Atmodjo et al., 2011; Purushotham et al., 2020; Zabotina et al., 2021). The CeSA proteins, homologs to CSLCs, form large complexes known as cellulose synthesizing rosettes, synthesizing 18–24 glucan chains in close proximity to allow for twisting and assembly into large microfilaments (Purushotham et al., 2020). In the case of XyG, when CSLC4 was recombinantly expressed in *Pichia pastoris*, together with XXT1, it produced long insoluble oligomers of β-(1- > 4) glucan. While CSLC4 expressed alone only produced small soluble β-(1- > 4) glucan (Cocuron et al., 2007). These experiments confirmed that CLSC4 was responsible for XyG glucan backbone synthesis, and strongly indicated that CSLC4 requires the presence of XXTs, most likely within a multiprotein complex, to synthesize longer glucan chains of the glucan backbone. It is well known that glucan oligosaccharides are insoluble in aqueous solutions unless they are branched.

Chou et al. (2012, 2015) investigated the interactions among XyG synthesizing proteins (CSLC4, XXTs, MUR3, XLT2, and FUT1) utilizing bimolecular fluorescent complementation (BiFC) and coimmunoprecipitation (co-IP) with *Arabidopsis* protoplasts as an expression system. Additionally, *in vitro* pull-down assays of BL21 *Escherichia coli* cells expressed truncated GTs were used to further confirm the results from protoplasts, suggesting that XyG GTs indeed interact with one another (Chou et al., 2012, 2015). Only three proteins were shown to strongly interact with themselves, forming homodimers of CSLC4, XXT2, and FUT1. XXT1 has been confirmed to homodimerize at high concentrations required for crystallization, but *in vivo*, XXT1 dimers were not detected (Chou et al., 2012; Culbertson et al., 2018). Homodimers of MUR3 and XXT5 were not detected with BiFC. It was also shown that XXT2 competes with XXT5 homodimers, reducing the levels of XXT5 homooligomerization *in vivo*. However, MUR3 homooligomerization was detected in another study using a Renilla luciferase complementation assay in *N. benthamiana* (Lund et al., 2015). Interactions with the CSLC proteins are likely central to anchoring GTs together to form the complex, as BiFC experiments show heterocomplexes of CSLC4-MUR3, CSLC4-XLT2, CSLC4-FUT1 and both XXT2 and XXT5 formed (Chou et al., 2012, 2015). CSLC4s’ distribution throughout all Golgi cisternae and interactions with other GTs provide a plausible indication that CSLCs recruit the other GTs to form a larger complex. Other interactions detected *via* both BiFC fluorescence and Renilla luciferase complementation included the XXT1-XXT2, XXT1-XXT5, XXT2-XLT2, and XLT2-FUT1 heterodimers (Chou et al., 2012, 2015; Lund et al., 2015). Additionally, pull-down assays confirmed the strong interactions between truncated variants of XXT2-XXT5, XXT2-FUT1, XXT5-FUT1, and MUR3-FUT1 expressed in *E. coli*, suggesting that GTs possibly interact *via* both their TMDs and catalytic domains localized to the Golgi lumen (Chou et al., 2012, 2015).

The types of interactions and how they may compete with one another among the different GTs within the multiprotein complexes have been proposed. Co-IP assays had demonstrated that homodimerization of XXT2 and FUT1 may be held together by disulfide bonds, as in reducing conditions, both proteins were exhibited in monomeric form, whereas homodimers were detected under non-reducing conditions (Chou et al., 2012, 2015). On the other hand, heterodimer XXT2-XXT5 is likely formed through non-covalent interactions, as XXT5 was only detected as a monomer (Chou et al., 2012, 2015). Alternatively, the crystal structure of XXT1 suggests another hypothesis, as XXT1 and XXT2 share the same amino acids involved in dimer formation. XXT5 has one mutation of an amino acid involved in the XXT1 homodimer, but shares the other amino acids involved in dimerization. It is likely that the XXT2-XXT5 dimer is formed *via* non-covalent interactions involving the same residues observed in the crystal structure of the XXT1 homodimer. This notion is supported by BiFC, Co-IP assays, and results from reverse genetics, as XXT2-XXT5 was the most stable and preferred form of both XXT2 and XXT5 (Chou et al., 2012).

Although compelling evidence shows dimer formation, the larger multiprotein complexes have yet to be studied. The interactions of two proteins between multiple combinations provide reasonable evidence that they likely interact with more than one at a time. Combined with localization evidence, the GTs likely interchange throughout different cisternae. It is highly likely these multiprotein complexes are not required for GT catalytic activity, as many of them are active alone in solution in *in vitro* assays (Culbertson et al., 2016, 2018; Rocha et al., 2016; Urbanowicz et al., 2017; Zhong et al., 2018, 2020, 2021; Ehrlich et al., 2021). In context to complexes, the formation of multiprotein structures likely increases their efficiency and the overall rate of XyG synthesis. Spatial consolidation of GTs activities together, rather than the random binding of freely moving XyG molecules between individual proteins on one hand, supports fast and reproducible synthesis of completely branched XyG molecules and, on another hand, prevents termination of reaction due to low solubility of the long unsubstituted glucan chains. The current hypothesis suggests that XyG synthesizing multiprotein complex in *Arabidopsis* is composed of a CSLC homodimer, with at least three XXTs (1, 2, and 5), both GalTs, and a Fut1 dimer.

Due to high homology, GTs such as AraTs and GalATs likely interact similarly within such complexes, with recombinant AraT replacing GalT in the *Arabidopsis* protein complexes. It is currently unknown how AceTs interact with XyG, but given the current evidence, acetylation likely occurs after fucosylation (Zhong et al., 2020). Therefore, it is reasonable to suggest that AceTs may not be involved in this complex. However, the acetylation of the glucan backbone may be necessary to increase the solubility of XXGG type XyG. The potential competition for the third Glc in the XXGG repeat indicates that these AceTs likely interact closely with the other GTs, but this is still only hypothetical and requires further investigation (Liu et al., 2016; Zhong et al., 2018). There is still much to be understood in how XyG synthesizing GTs interact with one another, and current information is insufficient to completely reveal the mechanism of XyG biosynthesis.

## Conclusion

This review presents the current state of knowledge about XyG biosynthesis. XyG synthesis is one of the most characterized polysaccharide biosynthesis processes in plants and serves as an excellent model for glycobiology by defining likely mechanisms and protein–protein interactions of the GTs involved in these processes. Most recent findings have focused on structural characterization, extending what is known about the protein structures and their homologs through numerous novel techniques that continue to evolve. Reverse genetics studies have elucidated numerous GTs and their effects on the plant’s cell wall, providing evidence for recent research focusing on enzyme structure and substrate specificity (Table 1). While a considerable amount of work has been done in elucidating XyG biosynthesis since the last reviews (Table 2), many aspects of this process are still unclear.

The regulation and quality control of XyG biosynthesis is still unknown. Regulation likely occurs either within the Golgi during synthesis of XyG, or post-synthetically in the apoplast. Golgi localized regulation likely occurs through the concentration of donor substrates and activity of synthesizing GTs. Low concentrations of either molecules could explain the variety of branching of *Arabidopsis* XyG found in the cell walls, such as XXG, XLXG/XXLG, XLLG, and XLFG. Alternatively, hydrolases within the plant cell wall may cleave mature XyG branches after its incorporation into cell wall, such as the fucosidase AXY8 and other less characterized hydrolases. Another interesting question to understand is how XyG structures are synthesized with high-fidelity. Since the promiscuity of XXTs and FUT1 was observed, what prevents the promiscuous binding of “incorrect” donor substrates in the Golgi is still unknown. Wide variations in the XyG monosaccharide composition are not detected in wild-type *Arabidopsis* XyG, suggesting the promiscuity observed in *in vitro* reactions is not observed *in vivo*. Although structural constraints of GTs are likely the most effective mechanism, these constraints clearly do not completely prevent binding of different substrates when natural donor substrates are absent, as observed in the *mur2* plants. Further research is required to understand how reproduction of the same structural pattern with high-fidelity is controlled and at what stage, either during synthesis, post-synthetically or, most likely, both.

Unfortunately, only two GTs involved in XyG biosynthesis have been structurally characterized to date. Due to significant advances in bioinformatics, reasonably accurate structural predictions of any GT are available *via* advanced software such as AlphaFold. However, structural characterization using biophysical techniques, such as X-ray crystallography or cryo-EM, is still required because prediction software is far from reliably predicting protein folding, particularly when dealing with TMDs and random coiling (stem regions) of GTs. Furthermore, the understanding of details in enzyme-substrate binding to provide accurate description of the catalytic mechanisms of GTs still requires biophysical methods.

Another largely under-investigated aspect of XyG biosynthesis is the understanding of protein–protein interactions and complex formation. Structures of FUT1 and XXT1 provided new hypotheses for understanding the mechanisms of FUT1 and XXT1 catalysis using their 3D structures. Structural studies of GTs were slow before due to their low expression, potential glycosylation, and tendency to oligomerize or even aggregate. New recent developments in using HEK293 cells and advances in sensitivity and accuracy of biophysical techniques allow for faster progress towards revealing structural organizations not only of single GTs but also their complexes. Co-expression of GTs in heterologous cells, utilization of artificial liposomes, nanodiscs, or more recently proposed amphipathic polymers together with high-resolution cryo-EM technology open new capabilities in advancing the studies of protein complex quaternary structures and the functions of GTs within such complexes.

While a considerable amount of work has been done in elucidating XyG biosynthesis in the last 5 years, there is much more to learn at the genetic, protein, and multiprotein level. To fully understand polysaccharide biosynthesis in plant Golgi, future protein work is urgently needed to elucidate GT mechanisms of enzymatic activity and substrate specificity, their localization and ER-Golgi transport, and their structural organization not only as an individual protein but, most importantly, their complexes.

## Author Contributions

All authors listed have made a substantial, direct, and intellectual contribution to the work and approved it for publication.

## Funding

This research was funded by a grant from the National Science Foundation (NSF), grant number NSF-MCB #1856477.

## Conflict of Interest

The authors declare that the research was conducted in the absence of any commercial or financial relationships that could be construed as a potential conflict of interest.

## Publisher’s Note

All claims expressed in this article are solely those of the authors and do not necessarily represent those of their affiliated organizations, or those of the publisher, the editors and the reviewers. Any product that may be evaluated in this article, or claim that may be made by its manufacturer, is not guaranteed or endorsed by the publisher.

## Footnotes

^1^ http://www.cazy.org/

^2^ https://www.uniprot.org
